# Habitat and seasonality shape the structure of tench (*Tinca tinca* L.) gut microbiome

**DOI:** 10.1038/s41598-020-61351-1

**Published:** 2020-03-10

**Authors:** Tomasz Dulski, Krzysztof Kozłowski, Slawomir Ciesielski

**Affiliations:** 1 Department of Environmental Biotechnology, University of Warmia and Mazury in Olsztyn, Olsztyn, Poland; 2 Department of Ichthyology and Aquaculture, University of Warmia and Mazury in Olsztyn, Olsztyn, Poland

**Keywords:** Metagenomics, DNA replication, Nutrition

## Abstract

Tench (*Tinca tinca* L.) is one of the most valued species of the *Cyprinidae*. This species is commercially important and has been intensively domesticated in recent years. To avoid excessive production losses, the health of farm fish must be maintained. Characterization of the tench gut microbiome can help achieve this goal, as the gastrointestinal microbiome plays an important role in host health. As part of this characterization, investigating the influence of the environment and season will help to understand the interrelationship between host and gut microbiota. Therefore, our aim was to use high-throughput 16S rRNA gene amplicon sequencing to profile the gut microbiome of tench. We studied two populations in summer and autumn: wild tench living in a lake and tench living in a pond in a semi-intensive fish farm. We found that, in the gut microbiome of all fish, the most abundant phylum was *Proteobacteria*, followed by *Firmicutes*, *Bacteroidetes* and *Actinobacteria*. Together, these phyla constituted up to 90% of the microbial communities. The abundance of *Candidatus Xiphinematobacter* differed significantly between lake and pond fish in summer, but not in autumn. In pond tench, *Methylobacterium* abundance was significantly lower in summer than in autumn. Mean Shannon, Chao1 indices and observed OTU’s indicated that microbial biodiversity was greater in the gut of lake fish than in that of pond fish. Beta-diversity analysis showed significant divergence between groups with both weighted and unweighted UniFrac distance matrices. Principal coordinates analysis revealed that more of the variance in microbial diversity was attributable to environment than to season. Although some of the diversity in lake tench gut microbiota could be attributable to feeding preferences of individual fish, our results suggest that environment is the main factor in determining gut microbiome diversity in tench.

## Introduction

Tench (*Tinca tinca* L.) is one of the most valued species of *Cyprinidae* fish and has great commercial importance^1^. Tench is generally considered to be one of the original European cyprinid species, which most likely evolved from primitive Tertiary *Paleoleuciscus* in large lake systems of Central Europe^2^. This species plays important roles in the environment, local economies and research. Tench provide many benefits to their ecosystems, effectively preventing blooms of algae, and recirculating minerals and nutrients that are deposited on the bottom of lakes and streams when they stir up the muddy bottoms in search of food^3^. This behavior helps reduce eutrophication. In Europe, tench are utilized as food, as ornamental fish and for leisure purposes such as angling, and have been used as an indicator of water quality in the context of fish assemblage^2^. In several European countries, tench have been reared in farm ponds, either in monoculture or alongside common carp (*Cyprinus carpio* L.). In recent years, tench have undergone intensive domestication, similar to that of common carp centuries ago. Due to their flavor, the production of tench for consumers is increasing, which may, in the near future, lead to rearing of this species in recirculating aquaculture systems^4^.

Over the past decades, research has shown that the gut microbiota play a key role in the health and nutrition of the host^5–7^. Fish gut microbiota contribute to digestion and can affect growth, reproduction, overall population dynamics and the vulnerability of the host fish to disease^8^. Much research involving comparative analysis of the microbiome of fish of the same species has shown that factors like environment and diet are two of many factors that influence the structure of the fish gut microbiota^9,10^. These factors may affect the microbiome by changing the relative abundance of individual groups of microorganisms. These changes can have repercussions on physiological, hormonal or cellular functions, which can result in the development of diseases. Research on the microbiome is particularly valuable in the domestication of fish because it reveals differences between the structure of microorganisms colonizing the digestive tract of wild animals and that of domesticated animals. This information can be used to properly compose feed and enrich it with appropriate probiotics and other supplements. Because the fish-gut microbiome is important for host health, it is generally accepted that identification of the gastrointestinal microbiota is undoubtedly important for understanding the functional interactions between the microbes and the host^11^. Although there have been some studies on the gastrointestinal microbiome of various fish species^10,12,13^, to the best of our knowledge, there is a lack of studies on the gut microbiome of tench (*T. tinca* L.).

Recently, thanks to access to mass sequencing techniques, it has become possible to get detailed information about the structure of the gut microbiome and its changes. Therefore, in this work we characterized the gastrointestinal microbiome of tench (*T. tinca* L.) using a mass sequencing approach based on gene coding for 16S rRNA. We compared the microbiomes of the gastrointestinal tracts of tench from a semi-intensive farm (pond) and those from a natural body of water (lake). Moreover, we examined the influence of seasonality on the fish gut microbiome by analysis of fish caught in Autumn and Summer. Information concerning the influence of habitat and seasonality on the fish gut microbiome may help to understand how the microbiome is affected by these factors, which microorganisms are dominant and which are most beneficial for the host. Moreover, it can be used to enhance the economic benefits of aquaculture by supplementing the feed of farmed fish species with the necessary probiotics.

## Methods

### Fish and rearing conditions

This study was carried out on fish caught from a natural body of water – Kortowskie Lake and Pond. Kortowskie Lake is a flow lake with an area of 89.7 ha located in the south-western part of Olsztyn (N 53 °45′43″ E 20 °26′44″). The maximum depth of Kortowskie lake is 17.2 m with an average depth 5.2 m. The location of the pond from which fish were obtained is N 53 °57′46″ E 21 °5′47″. This pond is a mid-forest body of water with an area of 4 ha, a maximum depth of 2 m and an average depth of 1.1 m. In the pond, fry of the following species were co-cultured: tench (*T. tinca* L.), common crucian (*Carassius carassius* L.), carp (*Cyprinus carpio* L.) and grass carp (*Ctenopharyngodon idella*). In the growing season (May–September), the fish were fed twice a week with a mixture of grain (wheat, barley and maize) that weighed approx. 10% of the stocking weight.

### Sampling

In autumn 2017 and summer 2018, 25 farm fish from the pond and 38 wild fish from the lake were randomly caught. Next, fish were weighed and measured. The final mean body weight and body length are shown in Table 1. After measurements, fish were stunned and decapitated quickly and correctly. The ventral body surface was wiped with a paper towel to remove excess mucus. All instruments, surfaces and the exterior of each fish were treated with 70% ethanol to sanitize the skin surface, and instruments were flame-sterilized prior to dissection. The ventral body surface was dried with a paper towel to remove any remaining ethanol. After opening the body cavity, the entire gastrointestinal tract and its contents were aseptically removed from each individual fish. The gut content was obtained by squeezing it into sterile tubes, after which it was stored at −20 °C until analysis.

**Table 1:** Final mean body weight and body length of tench from different environments.

Group	Autumn 2017		Summer 2018	
	Final weight (g)	Length (cm)	Final weight (g)	Length (cm)
Pond	8.38 ± 0.44	6.88 ± 1.27	12.63 ± 6.70	9.88 ± 1.98
Lake	21.27 ± 9.94	11.36 ± 1.52	19.041 ± 12.618	10.63 ± 2.341

Values are given as mean value ± SD (n = 8 fish per each group).

An ethics statement is not required for this type of research. No specific permissions were required for the described studies. The habitats of the fish are not protected in any way and they do not contain endangered or protected species. Fish from Kortowskie Lake were caught with the permission of the local authorities (Faculty of Environmental Sciences, University of Warmia and Mazury in Olsztyn). Fish from the pond were captured with the permission of co-author KK, an owner of the pond.

### DNA extraction

For isolation, we weighed around 100 mg gut content of each fish. Before isolation, samples of gastrointestinal contents were homogenized using plastic spatulas. Metagenomic DNA extractions were then performed with a QIAmp DNA Stool Mini Kit (Qiagen, Germany) following the manufacturer’s instructions. Qubit 2·0 Fluorometer (Invitrogen, Poland) was used to obtain accurate DNA quantification. The integrity of each DNA sample was assessed using 1% agarose gel electrophoresis. The purified DNA was suspended in 60 𝜇l of elution buffer and stored at −20 °C.

### 16S rRNA gene amplicon library preparation and sequencing

To check the quality and to be sure that the isolated DNA belongs to bacteria, we performed a PCR reaction using two universal 16S rRNA primers: 8 F and 534R^14^. Based on the results from PCR amplification and DNA yield we choose 32 samples from four groups (8 per group: Lake_Autumn_2017; Pond_Autumn_2017; Lake_Summer_2018; Pond_Summer_2018) for mass sequencing of 16S rRNA amplicons, which was done by an outside company (Genomed S.A, Poland). The Illumina protocol “16S Metagenomic Sequencing Library Preparation” was applied to prepare the 16S rRNA gene amplicons for the Illumina MiSeq system. The variable V3 and V4 regions of the 16S rRNA gene were amplified from bacterial DNA obtained from fish gut content samples. The PCR reactions were performed using 16S rRNA forward (5′ CCTACGGGNGGCWGCAG 3′) and reverse primers (5′ GACTACHVGGTATCTAATCC 3′) that were given by Klindworth *et al*.^15^. PCR amplification was performed accordingly to Illumina protocol. Amplicons were indexed using Nextera®XTIndex Kit accordingly to producer’s instructions. DNA was sequenced on an Illumina MiSeq instrument using 2 × 250 paired-end protocol. For sequencing, a Miseq Reagent Kit v3 (Illumina, San Diego, USA) was used for library sequencing.

### Sequencing data analysis

Raw paired-end sequences (3,620,064 reads from 32 samples) were processed using the QIIME 2^16^ software package (https://qiime2.org; version: 2018.8). Using Qiime 2 software, paired-end sequences were merged. This step reduced the 3,620,064 reads to 3,383,070 reads. Reads that software could not merge were excluded from further analyses. Next the data sequences underwent quality control using Deblur plugin in Qiime 2^17^. Deblur uses sequence error profiles to associate erroneous sequence reads with the true biological sequence from which they are derived, resulting in high quality sequence variant data. Quality control was performed in two steps. First, an initial filtering process quality score (q = 20) was applied^18^. This step removed 539,889 reads (15.96% data). Second, Deblur workflow was applied. In this step based on the median quality score, all reads were trimmed to 435 bp length. Also chimeric sequences were detected and excluded from analyses. 16S rRNA OTUs were picked from the Illumina reads using a closed-reference OTU picking protocol against the Greengenes database (https://docs.qiime2.org/2018.8/data-resources; data files: 13_8) clustered at 97% identity and trimmed to span only the 16S rRNA V4 region flanked by sequencing primers 515F-806R. Taxonomy assignments were associated with OTUs based on the taxonomy associated with the Greengenes reference sequence defining each OTU. Out of the 283,854 Illumina reads from the V4 region of the bacterial 16S rRNA genes that passed the QIIME quality filters, 53.3% (151,382 reads) matched a reference sequence at 97% nucleotide sequence identity. Next, OTU counts were binned into genus-level taxonomic groups for plots preparation.

Sequencing data were exported as individual fastq files and have been deposited in Sequence Read Archive (SRA) NCBI (https://www.ncbi.nlm.nih.gov/) as Bioproject under the accession code: PRJNA542255.

### Statistical analysis

Normality and homogeneity of variance of all weight and length data of fish were checked by Shapiro-Wilk’s and Levene’s test, respectively, using STATISTICA v.13.1 (StatSoft, Inc). To get a reliable statistical analysis, we rejected 4 samples with less than 1483 reads (3 from Lake_Autumn_2017 and 1 Pond_Autumn_2017) from the analysis due to the low number of reads assigned to taxon levels. The number of reads across samples was normalized by sample size and the relative abundance (%) of each taxon was calculated. All taxa present in the gut microbiome were considered for statistical analysis. Statistical analysis of intestinal microbial profiles was performed using the Statistical Analysis of Metagenomics Profiles (STAMP) program (http://kiwi.cs.dal.ca/Software/STAMP), retaining unclassified reads^19^. P-values were calculated by ANOVA followed by Tukey-Kramer post-hoc test and corrected for multiple comparisons using the Benjamini-Hochberg method for a False Discovery Rate (FDR) of 5%^20^. Furthermore, t-tests was used to check statistical differences between fish with extreme values of body weight and length in each group.

Alpha- and beta-diversity statistics were performed using QIIME 2 scripts diversity plugin, which supports metrics for calculating and exploring community alpha- and beta-diversity through statistics and visualizations in the context of sample metadata. In the calculation of alpha-diversity metrics, the normalization was performed using the “rarefaction” QIIME 2 process with standard parameters setting the max_rare_depth (upper limit of rarefaction depths) to mean sample size. Alpha-diversity metrics were calculated using ‘observed species’, ‘Chao1 index’ (species richness estimator), ‘Shannon’s diversity index’ and ‘Good’s coverage’. The alpha-diversity values at the same rarefaction level were calculated.

Differences in the beta-diversity of bacterial communities were verified using nonparametric Permutational Multivariate Analysis of Variance (PERMANOVA) test with 999 permutations. A pairwise significance test was also performed comparing groups from different sampling times and environments using the same distance matrix metrics (weighted and unweighted UniFrac distances). These tests were available in QIIME 2.

Beta-diversity metrics are an estimate of between-sample diversity of microbial profile and they were calculated by QIIME 2 “diversity beta-group-significance” script. We used both weighted (presence/absence/abundance matrix) and unweighted (presence/absence matrix) UniFrac distances^21,22^. The distance matrices were graphically visualized by three-dimensional PCoA representations.

## Results

### Qiime analysis of sequencing data

The sequence fastq files from the Illumina MiSeq were analyzed using QIIME 2 software. After filtering for quality, trimming length, and assigning taxonomies, the number of reads taxonomically classified according to the Greengenes database was 150,761 (Table 2). This value corresponded to an average number of 5384 ± 3298.33 per sample (range 1483–14,984). We identified 587 OTUs at 97% nucleotide sequence identity in tench gut content samples. After rarefaction, normalizing to the sample with the mean number of reads (4500), the observed species number per sample was between 7 and 89, corresponding to average number of counts per group between 31 and 49 (Table 2). Good’s coverage values for all groups were ≥ 0.99, indicating that sequencing coverage was attained and that the OTUs found in the samples were representative of the sampled population (Table 2). Although we observed that the mean values of observed species as well as Shannon diversity index (which reflects both the abundance and evenness of the species present) differed between environments (Table 2), these differences were not statistically significant. However, analyzing statistically the Chao1 index of the examined groups, we found that environment was a factor which affected species richness (P = 0.000036). Seasonality did not significantly affect the indices used in this study.

**Table 2:** Mean number of reads per sample assigned to OTUs, and alpha-diversity metrics values of gut microbial community of tench from different environment.

Group	Reads	Observed species	Shannon	Good’s coverage	Chao1
**Autumn 2017**					
Pond	3654 ± 1203.31	35 ± 13.39	3.44 ± 1.07	0.99 ± 0.01	41.14 ± 16.11
Lake	4256 ± 2887.29	49 ± 28.29	4.17 ± 1.47	0.99 ± 0.01	80.3 ± 34.21
**Summer 2018**					
Pond	6552 ± 3977.29	31 ± 17.79	3 ± 1.40	0.99 ± 0.01	52.33 ± 17.41
Lake	6436 ± 3661.96	44 ± 17.51	3.6 ± 0.94	0.99 ± 0.01	95.36 ± 22.82
Total number of reads taxonomically classified				150,761	
Mean number of reads per sample				5384 ± 3298.33	
Total number of OTU’s				587	

**Figure 1:**
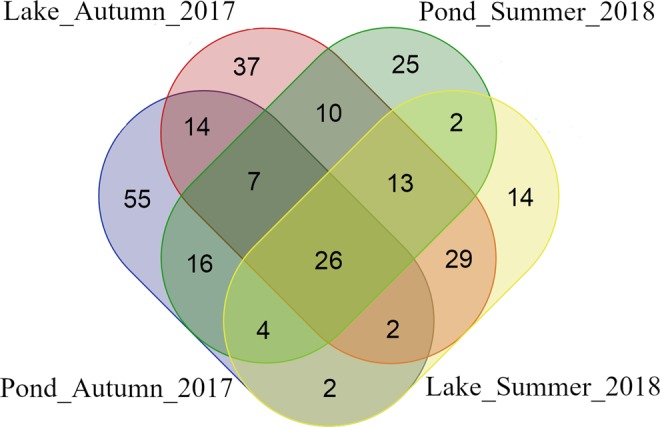
Numbers of common and unique OTUs present in the four groups of tench.

A Venn diagram (Fig. 1) was constructed to visualize OTUs common to each group of fish and present in the gut microbial community regardless of investigated factors and to find those unique to each specific group. The number of common OTUs presented in all groups was 26 and unique OTUs for each group varied from 14 to 55 (Fig. 1). List of the common and unique OTUs present in groups was reported in Supplementary Material (Table S1).

### Gut microbiome of tench

**Figure 2:**
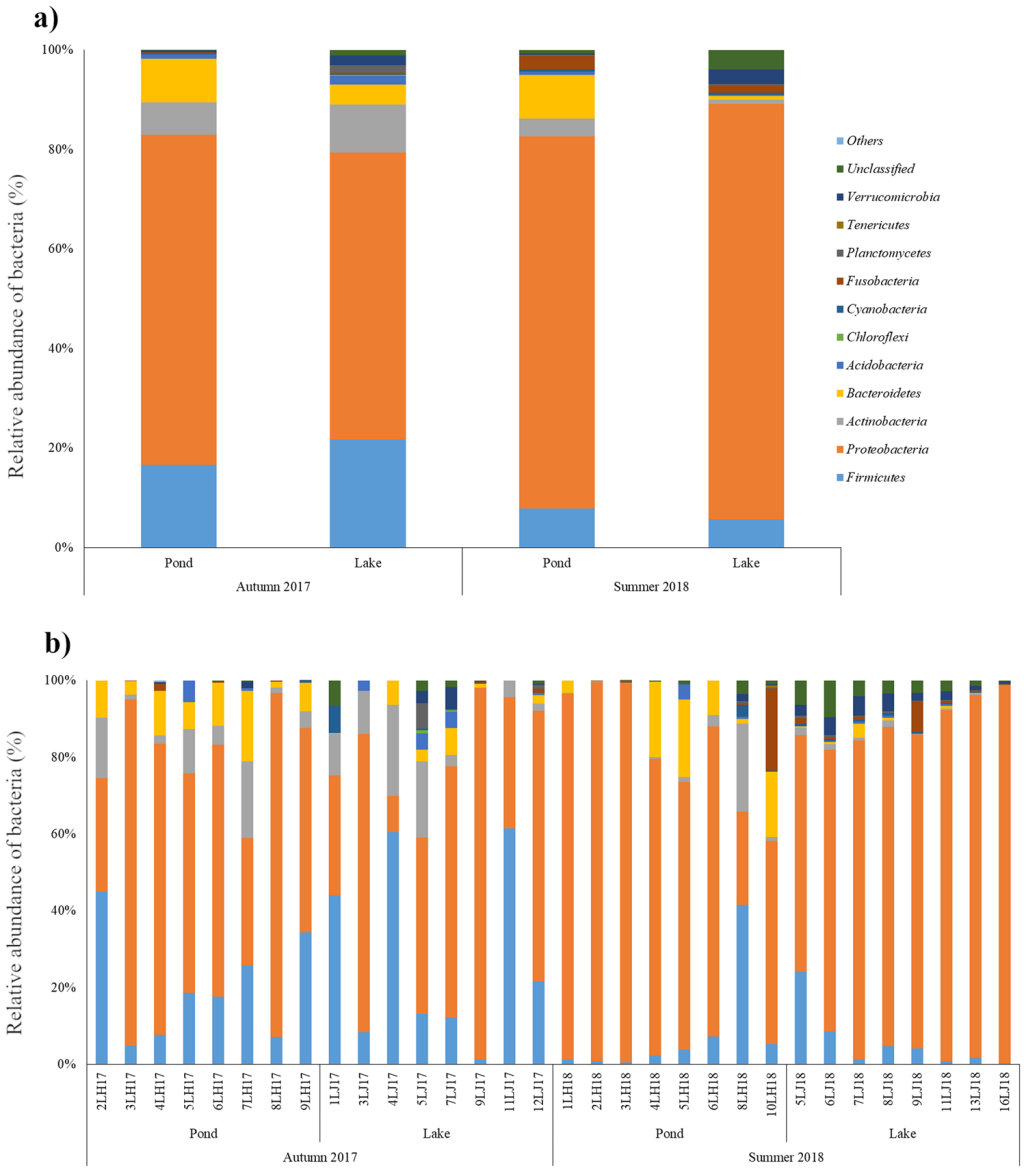
Relative abundance (%) of the overall most prevalent phyla in the different tench groups (**a**) and in individual fish (**b**). In the figures, all bacteria with an overall abundance >0,5% were reported. Bacteria with an abundance less than 0,5% were pooled and indicated as “Others”.

**Figure 3:**
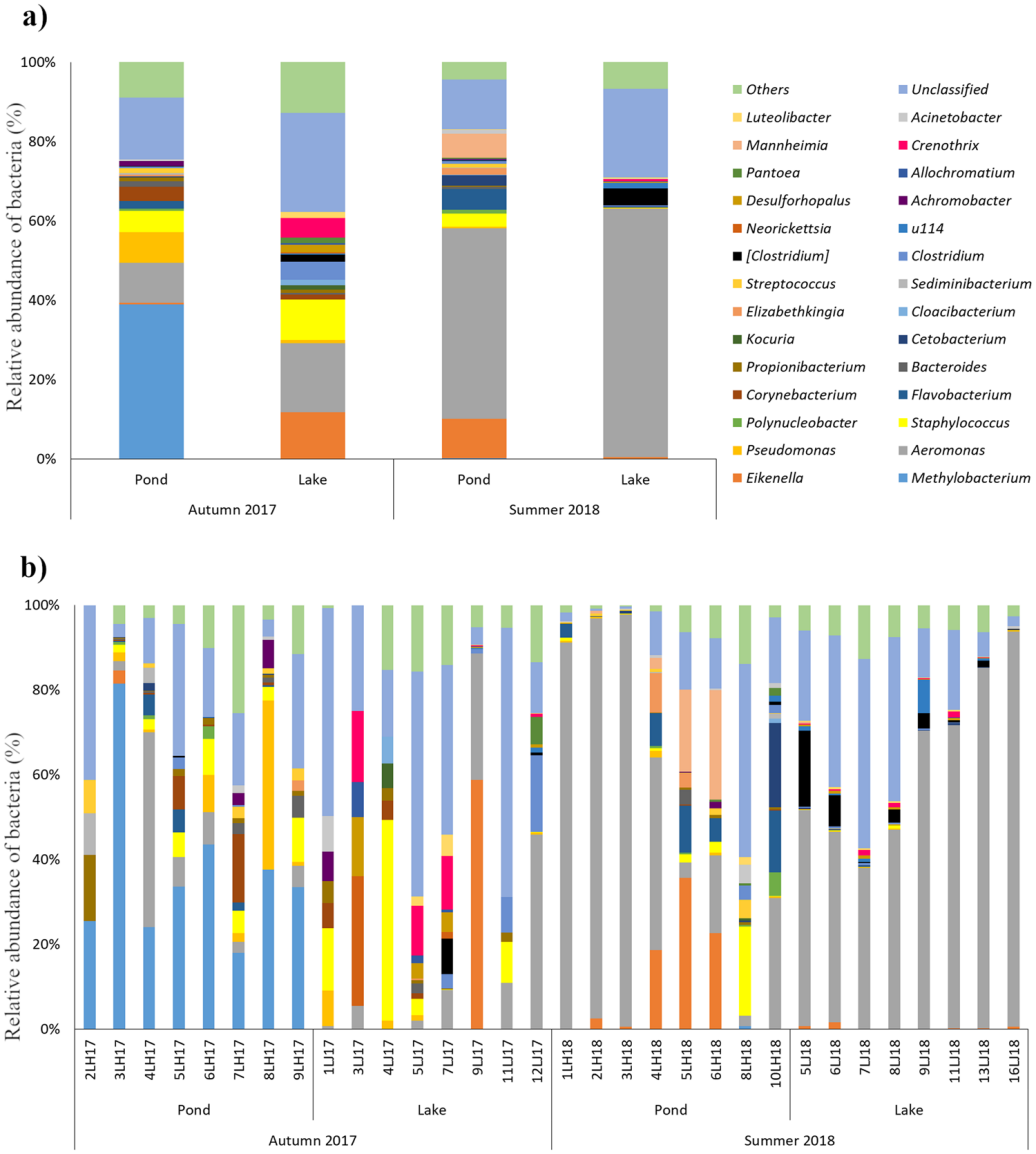
Relative abundance (%) of the overall most prevalent genera in the different tench groups (**a**) and in individual fish (**b**). In the figures, all bacteria with an overall abundance >5% were reported. Bacteria with an abundance less than 5% were pooled and indicated as “Others”.

The gut microbiome of 32 fish representing four groups divided depending on environment and season of sampling were examined to characterize their structure and to reveal the differences between them. We successfully described the microbiome structure of each investigated group of fish at the phylum and genus level, and classified 13 phyla and 125 genera. The gut microbial communities of each group and of individual fish are presented at the phylum (Fig. 2) and genus (Fig. 3) levels.

At the phylum level in each group, gut microbiota was dominated by the members of *Proteobacteria*, abundance on the mean level from 57.61% ±32.57 in Lake_Autumn_2017 to 83.49% ±11.90 in Lake_Summer_2018 (Fig. 2a). *Firmicutes* (5.65% ±7.95 to 21.72% ±22.89); *Actinobacteria* (0.86% ±0.72 to 9.71% ±11.22); *Bacteroidetes* 0.73% ±1.17 to 8.81% ±8.92) were less abundant (Fig. 2a). The bacterial community composition in individual fish was mostly similar to the mean phylum abundance in groups, except for fish 2LH17, 1LJ17, 4LJ17, 11LJ17, 8LH18, in which the gut microbiome was dominated by *Firmicutes* (45.10%, 44.06%, 60.55%, 61.40%, 40.52%, respectively) (Fig. 2b).

At the genus level, the gut microbiota of fish caught in summer 2018 was dominated by the members of *Aeromonas*, especially in Lake_Summer_2018 group (mean abundance of 62.61% ±20.25), where this genus dominated the gut microbiota of each fish (Fig. 3a,b). In contrast, in the fish catch in autumn, this genus was less abundant (from 10.05% ±16.05 in Pond_Autumn_2017 to 17.38% ±19.8 in Lake_Autumn_2017 (Fig. 3a). The gut microbiota of Pond_Autumn_2017 was dominated by the members of *Methylobacterium*, with a mean abundance of 38.88% ±20.6 of all bacteria at genus level, whereas in the other groups, we either did not observe any *Methylobacterium*, or in Pond_Summer_2018, we observed only a small amount (0.09% ±0.25) (Fig. 3a). In contrast, no genus clearly predominated in Lake_Autumn_2017. It is worth noting that, in fish 4LH18, 5LH18, 6LH18 and 9LJ18, we observed higher percent abundance of *Eikenella* (18.7%; 35.7%; 22;7%; 58.8% respectively), whereas in other fish they were less abundant, below 3%.

There were statistically significant differences between groups in terms of the abundance of some bacteria. Table 3 presents the relative abundance of phyla and genera that differed significantly between groups. The mean abundance of *Methylobacterium* differed significantly between groups (P = 0.00003), due to the high content of these bacteria in Pond_Autumn_2017. Also, the mean abundance of the genus *Candidatus Xiphinematobacter* differed significantly between groups (P = 0.00007), which made up a large portion of *Verrucomicrobia*, particularly in Lake_Summer_2018 (P = 0.02). The difference in mean abundance of unclassified *Xanthomonadaceae* was also significant (P = 0.04). Statistical analysis of all taxa were reported in Supplementary Table S2.

**Table 3:** Mean relative abundance (%) ± SD of phyla and genera that were significantly different between groups.

	Autumn 2017				Summer 2018				p-values (corrected)	Effect size
	Pond		Lake		Pond		Lake			
	mean relative abundance (%)	± SD	mean relative abundance (%)	± SD	mean relative abundance (%)	± SD	mean relative abundance (%)	± SD		
***Phylum***										
*Verrucomicrobia*	0.32	0.59	1.92	2.26	0.28	0.62	2.91	1.6	0.02677	0.42
***Genus***										
*Methylobacterium*	38.88	19.08	0.0	0.0	0.09	0.24	0.0	0.0	0.00003	0.76
*Candidatus Xiphinematibacter*	0.01	0.03	0.03	0.04	0.05	0.13	2.00	1.02	0.00007	0.72
*Unclass. Xanthomonadaceae*	0.0	0.0	0.03	0.03	0.0	0.0	1.27	1.1	0.04	0.49
*Unclass. Bacteria*	0.09	0.14	1.10	0.98	0.72	1.13	3.93	2.7	0.04	0.48

Statistical Analysis of Metagenomics Profiles (STAMP) software was used to test statistical significance of each taxa between groups abundances, unclassified reads were retained only for calculating frequency. One-Way ANOVA (P < 0.05), with an effect size (ETA-Squared) with Benjamini-Hochberg FDR correction was applied followed by Tukey-Kramer post-hoc test. The result of all analysis is reported in Supplementary Table S2.

The results of post hoc multiple comparisons are shown in Table 4. Significant statistical differences of mean abundance of taxa were observed only between Pond_Summer_18 vs Lake_Summer_18 and Pond_Autumn_17 vs Pond_Summer_18 (Table 4).

**Table 4:** Results of the post-hoc test of differences in mean abundance of the phyla and genera with Benjamini-Hochberg FDR correction of P-values.

	Between environment		Between Season	
	Pond_Autumn_17 vs Lake_Autumn_17	Pond_Summer_2018 vs Lake_Summer_2018	Pond_Autumn_17 vs Pond_Summer_18	Lake_Autumn_17 vs Lake_Summer_18
	P value (corrected)	P value (corrected)	P value (corrected)	P value (corrected)
***Phylum***				
*Verrucomicrobia*	>0.05	0.0166	>0.05	>0.05
***Genus***				
*Methylobacterium*	>0.05	>0.05	0.03	>0.05
*Candidatus Xiphinematibacter*	>0.05	0.04	>0.05	>0.05

**Figure 4:**
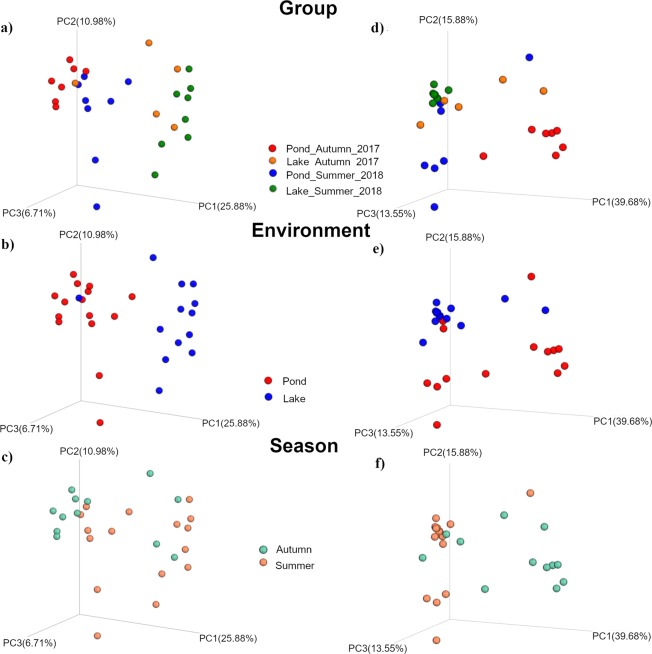
Beta-diversity metrics. Principal coordinate analysis (PCoA) of Unweighted (**a**–**c**) and Weighted (**d**–**f**) Unifrac distances of gut microbial communities associated to different environment and season. The figures show the plot of individual fish according to their microbial profile.

QIIME 2 was used to compute microbial beta-diversity metrics. Analyses were performed using weighted and unweighted UniFrac distances. Data of UniFrac metrics was used to prepare three-dimensional plots using principal coordinates analysis (PCoA) (Fig. 4). Weighted and unweighted PCoA showed that all samples in Pond_Autumn_2017 were clustered together, and all of those from Lake_Summer_2018 were in a separate cluster. Most of samples in Lake_Autumn_2017 and Pond_Summer_2018 were scattered, nonetheless some of them were more clustered with Lake_Summer_2018 group (Fig. 4a,d). The PCoA plots suggest that both factors may have influenced the fish gut microbiome in all examined groups. However, environment seems to affected more on the fish gut microbiome than season. In Fig. 4(b,e) groups from the same environments clustered together more than dots on the season plots (Fig. 4c,f), where samples were less clustered and were scattered.

The statistical analysis (permutation multivariate analysis PERMANOVA) reflected the PCoA plot results, indicating a significant divergence between groups for both weighted (P = 0.001; Pseudo-F = 4.71) and unweighted (P = 0.001; Pseudo-F = 3.37) UniFrac distance matrices (Table 5). Result of pairwise test on unweighted and weighted UniFrac data revealed, that all group were significantly diverged (Table 5).

**Table 5:** Permutation multivariate analysis PERMANOVA test on weighted and unweighted Unifrac data of intestinal microbiomes of tench living in different environments.

PERMANOVA analysis	Unweighted Unifrac		Weighted Unifrac	
	P-value	Pseudo-F	P-value	Pseudo-F
**One-way**				
All groups	0.001	3.37	0.001	4.71
**PERMANOVA pairwise test:**				
Pond_Autumn_2017 *vs*	Pond_Summer_2018	0.004		0.002
Pond_Autumn_2017 *vs*	Lake_Autumn_2017	0.011		0.001
Pond_Summer_2018 *vs*	Lake_Summer_2018	0.001		0.022
Lake_Autumn_2017 *vs*	Lake_Summer_2018	0.013		0.013

## Discussion

Recent studies of the fish-gut microbiota of many species have focused on examining how environmental factors (e.g. diet, habitat) affect microbial communities and how changes in these factors influence the gut microbiome^23,24^. Since environmental factors are thought to play a role in shaping the intestinal microbiota of fish, we hypothesized that the gut microbiota of tench living in a semi-intensive pond differs from that of tench living in a lake, and that the gut microbiota will change across the seasons. Our results provide information on the gut microbiota of tench and highlight associations between environmental factors and gut microbiota. An understanding of these associations provides information that may be useful for addressing problems during the domestication of these valuable freshwater fish.

Our study indicated that, in the tench gut microbiome, the phyla *Proteobacteria* and *Firmicutes* predominate, followed by lesser percentages of *Bacteroidetes* and *Actinobacteria* (Fig. 2). This observation suggests that these phyla play valuable roles in the health and digestion of tench, based on three lines of reasoning. First, it is established that specific bacteria species in the gut microbiota are important for health and digestion^25^. For example, Ray *et al*.^26^ report numerous examples of amylase, protease-, lipase-, chitinase-, cellulose and phytase-producing bacteria isolated from the gastrointestinal tract of fish. Second, although it is difficult to estimate the contribution of specific bacteria to the function of the whole gut ecosystem, it is reasonable to expect that the overall gut microbiome will be strongly influenced by the predominant microorganisms^27^. Third, the presence of similar bacterial taxa in the gut microbiota of multiple fish species suggests that these bacteria are valuable for the host and could play important roles in digestion, nutrient absorption and immune response^8^. *Proteobacteria*, *Firmicutes*, *Bacteroidetes* and *Actinobacteria* have been found in the intestines of many marine and freshwater fish species^9,10,28–30^ and other *Cyprinidae* species^31^. Moreover, a number of studies of fish have found that these phyla are present in proportions similar to those in our study^24,32,33^. Thus, our results suggest that *Proteobacteria*, *Firmicutes*, *Bacteroidetes* and *Actinobacteria* may play important roles in the health and digestion of tench.

The structure of the gut microbiome differs between many species of fish. This mainly depends on external factors. However, some species might be unique to different hosts due to evolutionary factors and host genetics. Figure 1 presents 26 common OTUs that were always present in the tench gut microbiome regardless of changes in external conditions. Most of them belonged to *Proteobacteria* and *Firmicutes* (Table S1), which is consistent with other results of this study. One genus found in almost all examined fish was *Aeromonas*. A study conducted on another *Cyprinidae* species (grass carp) showed that *Aeromonas* dominated the gut microbiome^34^. Although their percent abundance was lower, *Clostridium* and *Bacteroidetes* were also present in all examined groups; these taxa have also been found in other herbivorous carps^31^. It is interesting to note that all these bacteria are widely known as cellulose-degrading bacteria, which are particularly important for food degradation in the gut of herbivores^31^. Thus, these findings suggest that these bacteria co-create the main stem of the *Cyprinidae* microbiome and are necessary to break down nutrients in digestive-tract contents.

The environment is one of the factors that affects the gut microbiota of fish^35^, and in our study, beta-diversity analysis based on the PERMANOVA test found statistically significant differences between groups from different environments (Table 5). This finding is consistent with that of a previous study on freshwater and marine fish^7^. In addition, differences in gut microbiota in various water habitats were observed in other *Cyprinidae* species related to tench, like grass carp, common carp^36^, silver carp (*Hypophthalmichthys molitrix*) and bighead carp (*Hypophthalmichthys nobilis*)^9^. In our study, there were statistically significant differences between the Pond_Summer_2018 and Lake_Summer_2018 groups at alpha-diversity level in terms of the percent abundance of *Candidatus Xiphinematibacter*, a representative of *Verrucomicrobia* (Table 4). Although the percent abundance of these bacteria was low, it was greater in the wild fish guts (Supplementary Table S2). It is possible that greater percent abundance of these bacteria in wild fish may be a result of the more diverse food which can be found in their habitat. The diet of the pond tench was supplemented with a mixture of grain, whereas the lake tench could eat their natural food, which might be insect larvae, small crustaceans or small snails^37^. Tench also eat dead plants and animals. Despite these differences in diet, alpha-diversity did not show statistically significant differences in percent abundance between Pond_Autumn_17 and Lake_Autumn_17. This observation can be explained by availability of food in lake while autumn, which was worse in contrast to summer and thus, diet was poorer and the biodiversity of gut microbiota was lower (See Shannon and Chao1 index Table 2). It is also worth adding that cause of these differences may be the result of the activity of the tench, which decreases in autumn, so that they need less food than in summer. On the other hand, as can be seen in Table 2, the number of OTUs and the biodiversity of the gut microbiota were usually higher in wild fish from the lake than in the farmed pond-fish. A previous study by Alaş *et al*.^38^ showed that the gut microbiome of tench varies depending on the season. All these findings suggest that the microbiome of wild fish is more complex due to the wider variety of food available in the lake.

Season is one of the factors that can shift the gut microbiota. Several reviews have indicated that seasonal variation and temperature changes influence fish-gut microbial composition^39,40^. Changes in total bacterial abundance of gut microbiota have been reported between summer and autumn periods^41^. Hagi *et al*.^42^ reported that the intestinal lactic acid bacteria composition of four *Cyprinidae* varied with seasons. It was revealed that abundance of lactic acid bacteria depended on the water temperature. Differences due to season were also apparent in largemouth bass (*Micropterus salmoides*) and spotted gar (*Lepisosteus oculatus*)^43^. However, in that study, seasonal changes were greater on the skin microbiome, suggesting that the gut is able to harbor a relatively stable community composition despite seasonal influences^44^. Our experiments are consistent with previous studies indicating that season is a factor which influences the gut microbiome. Although we did not observed any changes in abundance of lactic acid bacteria, it is interesting to note that beta-diversity analysis showed that season significantly affected on the composition of the gut microbiota (Table 5). This difference was mainly due to statistically significant changes in the percent abundant of *Methylobacterium* (P = 0.00003) belonging to *Methylobacteriaceae* (Table 4). *Methylobacteriaceae* members were previously detected in other studies^45^, which indicate that this family of bacteria is a part of the fish gut microbiome. Although Larsen *et al*.^45^ found that *Methylobacteriaceae* comprised below 1% of the entire gut microbiome in three species, our study showed that the abundance of *Methylobacterium* could be almost 40% (Pond_Autumn_17 group, Fig. 3, Supplementary Table S2). The reason for the unexpectedly high percent abundant of *Methylobacteriaceae* is not clear, but it might be related to temperature of water. According to Givens (2007)^46^, higher temperature could increase the abundance of *Methylobacteriaceae* in the gut microbiome of *Lagodon rhomboids*. These findings are in contrast to our results, which showed a higher amount of *Methylobacteriaceae* in Autumn, when the temperature of water was lower than in Summer. Thus, the influence of water temperature should be interpreted with caution. Zarkasi *et al*.^47^, in their study on the influence of season on gut microbiota composition, suggested that the gut microbiome was not affected directly by water temperature, but rather by diet.

It could be hypothesized that factors like body weight and length may also affect the fish gut microbiota and influence its composition and diversity, similarly to the factors described in this paper. To exclude the influence of these factors, we took the extreme values of body weight and length in each group and analyze them. We did not found statistically significant differences between the gut microbiome composition of these fish, which indicates that the observed differences are due to the analyzed factors. However, because we analyzed fry from a pond and wild fish at various stages of development, ontogeny might also have influenced the fish gut microbiota, as has been previously reported^48^.

## Conclusions

To sum up, our research provides the first detailed description of the microbiome structure of tench (*T. tinca* L.) from different habitats. Alpha-diversity analysis based on the metrics used in our study found no statistically significant differences between the gut microbiome compositions of fish from different seasons. Only the environment had a significant effect on species richness. Analysis of percent abundance showed some significant differences between groups with respect to *Methylobacterium* and *Candidatus Xiphinematibacter*. Beta-diversity analysis on unweighted and weighted UniFrac showed statistically significant differences between each pair of groups that were compared. Both seasonality and environment had a significant influence on the microbiome structure. However, the environment had a stronger effect than seasonality, as is clearly shown in Fig. 4, where samples are clustered more tightly when classified by environment and less tightly when classified by season. However, the results of this study should be treated with caution. It cannot be excluded that ontogeny had an effect on gut microbiota structure. Nevertheless, future interspecific studies with large representative samples of individuals from different geographical areas and habitat types are needed to precisely define the role of these factors in shaping the gut microbial composition. Studies on fish gut microbiota may help to improve the welfare of fish and aquaculture practices. Future research on the specific functional role of these microorganisms within the tench gut microbiome is needed.

## Supplementary information

Supplementary materials.

## Data Availability

All fastq files obtained from sequencing are available from the NCBI Sequence Read Archive (SRA) repository, (https://www.ncbi.nlm.nih.gov/sra; Bioproject accession number PRJNA542255). Rest relevant data are within the manuscript and its Supplementary Files.
