# Supplementary material for: Habitat and seasonality shape the structure of tench (Tinca tinca L.) gut microbiome
Source: Sci Rep. 2020 Mar 10;10:4460. doi: 10.1038/s41598-020-61351-1 (PMC7064478; doi:10.1038/s41598-020-61351-1)
Supplement: Supplementary file 1 — Supplementary materials. [file 41598_2020_61351_MOESM1_ESM.pdf]

Habitat and seasonality shape the structure of tench (Tinca tinca L.) gut microbiome

Tomasz Dulski, Krzysztof Kozłowski, Slawomir Ciesielski

Table S1. Common and unique OTUs present in the four groups of tench.

Groups		Number of shared OTUs	Taxons
All groups	LakeAutumn2017 LakeSummer2018 PondAutumn2017 PondSummer2018	26	k__Bacteria; p__Firmicutes; c__Clostridia; o__Clostridiales; f__Clostridiaceae; g__Clostridium
			k__Bacteria; p__Firmicutes; c__Bacilli; o__Bacillales; f__Staphylococcaceae; g__Staphylococcus
			k__Bacteria; p__Firmicutes; c__Bacilli; o__Bacillales; f__Bacillaceae
			k__Bacteria; p__Proteobacteria; c__Gammaproteobacteria; o__Aeromonadales; f__Aeromonadaceae; g__Aeromonas; s__caviae
			k__Bacteria; p__Firmicutes; c__Bacilli; o__Lactobacillales; f__Enterococcaceae
			k__Bacteria; p__Proteobacteria; c__Betaproteobacteria
			k__Bacteria; p__Proteobacteria; c__Alphaproteobacteria; o__Rhizobiales
			k__Bacteria; p__Proteobacteria; c__Betaproteobacteria; o__Rhodocyclales; f__Rhodocyclaceae
			k__Bacteria
			k__Bacteria; p__Proteobacteria; c__Betaproteobacteria; o__Burkholderiales; f__Alcaligenaceae; g__Achromobacter; s__
			k__Bacteria; p__Firmicutes; c__Clostridia; o__Clostridiales; f__Clostridiaceae; g__Clostridium; s__gasigenes
			k__Bacteria; p__Actinobacteria; c__Actinobacteria; o__Actinomycetales
			k__Bacteria; p__Proteobacteria; c__Betaproteobacteria; o__Burkholderiales; f__Oxalobacteraceae; g__Polynucleobacter; s__
			k__Bacteria; p__Actinobacteria; c__Actinobacteria; o__Actinomycetales; f__Propionibacteriaceae; g__Propionibacterium; s__
			k__Bacteria; p__Proteobacteria; c__Betaproteobacteria; o__Burkholderiales; f__Comamonadaceae; g__Acidovorax
			k__Bacteria; p__Proteobacteria; c__Gammaproteobacteria; o__Pseudomonadales; f__Pseudomonadaceae; g__Pseudomonas
			k__Bacteria; p__Proteobacteria; c__Gammaproteobacteria; o__Enterobacteriales; f__Enterobacteriaceae
			k__Bacteria; p__Bacteroidetes
			k__Bacteria; p__Actinobacteria; c__Actinobacteria; o__Actinomycetales; f__Micrococcaceae; g__Micrococcus; s__
			k__Bacteria; p__Firmicutes; c__Clostridia; o__Clostridiales; f__Peptostreptococcaceae; g__[Clostridium]; s__bifermentans
			k__Bacteria; p__Actinobacteria; c__Actinobacteria; o__Actinomycetales; f__Corynebacteriaceae; g__Corynebacterium; s__variabile
			k__Bacteria; p__Fusobacteria; c__Fusobacteriia; o__Fusobacteriales; f__Fusobacteriaceae; g__u114; s__
			k__Bacteria; p__Proteobacteria; c__Betaproteobacteria; o__Burkholderiales; f__Comamonadaceae
			k__Bacteria; p__Verrucomicrobia; c__[Spartobacteria]; o__[Chthoniobacterales]; f__[Chthoniobacteraceae]; g__Candidatus Xiphinematobacter; s__
			k__Bacteria; p__Proteobacteria; c__Betaproteobacteria; o__Neisseriales; f__Neisseriaceae; g__Eikenella; s__
			k__Bacteria; p__Proteobacteria; c__Gammaproteobacteria; o__Aeromonadales; f__Aeromonadaceae; g__Tolumonas; s__

From the same season	LakeAutumn2017 PondAutumn2017	14	k__Bacteria; p__Firmicutes; c__Clostridia; o__Clostridiales; f__Veillonellaceae; g__Dialister; s__
			k__Bacteria; p__Actinobacteria; c__Actinobacteria; o__Actinomycetales; f__Geodermatophilaceae; g__; s__
			k__Bacteria; p__Firmicutes; c__Bacilli; o__Lactobacillales; f__Leuconostocaceae; g__Leuconostoc; s__
			k__Bacteria; p__Bacteroidetes; c__Bacteroidia; o__Bacteroidales
			k__Bacteria; p__Acidobacteria; c__Acidobacteria-6; o__; f__; g__; s__
			k__Bacteria; p__Proteobacteria; c__Betaproteobacteria; o__Neisseriales; f__Neisseriaceae
			k__Bacteria; p__Firmicutes; c__Bacilli; o__Lactobacillales; f__Streptococcaceae; g__; s__
			k__Bacteria; p__Firmicutes; c__Bacilli; o__Bacillales; f__Planococcaceae
			k__Bacteria; p__Proteobacteria; c__Gammaproteobacteria; o__Xanthomonadales; f__Xanthomonadaceae; g__Stenotrophomonas
			k__Bacteria; p__Proteobacteria; c__Epsilonproteobacteria; o__Campylobacterales; f__Campylobacteraceae; g__Arcobacter; s__cryaerophilus
			k__Bacteria; p__Proteobacteria; c__Gammaproteobacteria; o__Alteromonadales; f__[Chromatiaceae]
			k__Bacteria; p__Bacteroidetes; c__[Saprospirae]; o__[Saprospirales]; f__Chitinophagaceae; g__Flavihumibacter; s__
			k__Bacteria; p__Bacteroidetes; c__Bacteroidia; o__Bacteroidales; f__Prevotellaceae; g__Prevotella; s__copri
			k__Bacteria; p__Firmicutes; c__Bacilli; o__Lactobacillales; f__Lactobacillaceae; g__Lactobacillus
	LakeSummer2018 PondSummer2018	2	k__Bacteria; p__Proteobacteria; c__Gammaproteobacteria; o__Pasteurellales; f__Pasteurellaceae; g__Mannheimia; s__
			k__Bacteria; p__Proteobacteria; c__Betaproteobacteria; o__Neisseriales; f__Neisseriaceae; g__Chitinilyticum; s__aquatile
From the same environment	PondAutumn2017 PondSummer2018	16	k__Bacteria; p__Bacteroidetes; c__Flavobacteriia; o__Flavobacteriales; f__Flavobacteriaceae; g__Flavobacterium; s__frigidarium
			k__Bacteria; p__Bacteroidetes; c__Flavobacteriia; o__Flavobacteriales; f__Flavobacteriaceae; g__Flavobacterium
			k__Bacteria; p__Firmicutes; c__Clostridia; o__Clostridiales; f__Veillonellaceae; g__Veillonella
			k__Bacteria; p__Bacteroidetes; c__Flavobacteriia; o__Flavobacteriales; f__Cryomorphaceae
			k__Bacteria; p__Bacteroidetes; c__Flavobacteriia; o__Flavobacteriales; f__Flavobacteriaceae; g__Flavobacterium; s__succinicans
			k__Bacteria; p__Proteobacteria; c__Betaproteobacteria; o__Burkholderiales
			k__Bacteria; p__Proteobacteria; c__Alphaproteobacteria; o__Rhizobiales; f__Methylobacteriaceae; g__Methylobacterium; s__organophilum
			k__Bacteria; p__Cyanobacteria; c__Nostocophycideae; o__Nostocales; f__Nostocaceae; g__Cylindrospermopsis; s__
			k__Bacteria; p__Proteobacteria; c__Epsilonproteobacteria; o__Campylobacterales; f__Campylobacteraceae; g__Arcobacter
			k__Bacteria; p__Bacteroidetes; c__Cytophagia; o__Cytophagales
			k__Bacteria; p__Proteobacteria; c__Gammaproteobacteria; o__Pseudomonadales; f__Moraxellaceae; g__Acinetobacter; s__guillouiae
			k__Bacteria; p__Bacteroidetes; c__Flavobacteriia; o__Flavobacteriales; f__[Weeksellaceae]; g__Elizabethkingia; s__meningoseptica
			k__Bacteria; p__Firmicutes; c__Bacilli; o__Lactobacillales
			k__Bacteria; p__Proteobacteria; c__Alphaproteobacteria; o__Rhizobiales; f__Rhizobiaceae
			k__Bacteria; p__Firmicutes; c__Clostridia; o__Clostridiales; f__Lachnospiraceae; g__Roseburia; s__faecis
			k__Bacteria; p__Proteobacteria; c__Betaproteobacteria; o__Neisseriales; f__Neisseriaceae; g__Vogesella; s__

From the same environment	LakeAutumn2017 LakeSummer2018	29	k__Bacteria; p__Proteobacteria; c__Alphaproteobacteria; o__Rhodobacterales; f__Rhodobacteraceae
			k__Bacteria; p__Verrucomicrobia; c__[Spartobacteria]; o__[Chthoniobacterales]; f__[Chthoniobacteraceae]; g__Chthoniobacter; s__
			k__Bacteria; p__Proteobacteria; c__Gammaproteobacteria; o__Xanthomonadales; f__Xanthomonadaceae
			k__Bacteria; p__Proteobacteria; c__Gammaproteobacteria; o__Chromatiales
			k__Bacteria; p__Bacteroidetes; c__Flavobacteriia; o__Flavobacteriales; f__Flavobacteriaceae; g__Lutimonas; s__
			k__Bacteria; p__Actinobacteria; c__Actinobacteria; o__Actinomycetales; f__Nocardioidaceae; g__Pimelobacter; s__
			k__Bacteria; p__Actinobacteria; c__Actinobacteria; o__Actinomycetales; f__Mycobacteriaceae; g__Mycobacterium; s__celatum
			k__Bacteria; p__Proteobacteria; c__Betaproteobacteria; o__Rhodocyclales; f__Rhodocyclaceae; g__Azovibrio; s__
			k__Bacteria; p__Proteobacteria; c__Gammaproteobacteria; o__Chromatiales; f__Chromatiaceae; g__Allochromatium; s__
			k__Bacteria; p__Proteobacteria; c__Gammaproteobacteria; o__Methylococcales; f__Crenotrichaceae; g__Crenothrix
			k__Bacteria; p__Proteobacteria; c__Deltaproteobacteria; o__Desulfobacterales; f__Desulfobacteraceae
			k__Bacteria; p__Actinobacteria; c__Actinobacteria; o__Actinomycetales; f__Intrasporangiaceae; g__Phycicoccus; s__
			k__Bacteria; p__Proteobacteria; c__Deltaproteobacteria; o__Desulfobacterales; f__Desulfobulbaceae
			k__Bacteria; p__Proteobacteria; c__Deltaproteobacteria; o__Desulfobacterales; f__Desulfobulbaceae; g__Desulfobulbus; s__
			k__Bacteria; p__Proteobacteria; c__Gammaproteobacteria; o__Methylococcales; f__Crenotrichaceae; g__Crenothrix; s__
			k__Bacteria; p__Proteobacteria; c__Deltaproteobacteria; o__Syntrophobacterales; f__Syntrophorhabdaceae; g__; s__
			k__Bacteria; p__Verrucomicrobia; c__Verrucomicrobiae; o__Verrucomicrobiales; f__Verrucomicrobiaceae; g__MSBL3; s__
			k__Bacteria; p__Proteobacteria; c__Gammaproteobacteria; o__Methylococcales; f__Crenotrichaceae; g__Crenothrix; s__polyspora
			k__Bacteria; p__Planctomycetes; c__Planctomycetia; o__Pirellulales; f__Pirellulaceae; g__planctomycete
			k__Bacteria; p__Bacteroidetes; c__Bacteroidia; o__Bacteroidales; f__Porphyromonadaceae; g__Dysgonomonas; s__
			k__Bacteria; p__Actinobacteria
			k__Bacteria; p__Proteobacteria
			k__Bacteria; p__Proteobacteria; c__Gammaproteobacteria; o__Acidithiobacillales; f__; g__; s__
			k__Bacteria; p__Proteobacteria; c__Deltaproteobacteria; o__Desulfobacterales; f__Desulfobulbaceae; g__Desulforhopalus; s__
			k__Bacteria; p__Proteobacteria; c__Gammaproteobacteria; o__Pseudomonadales; f__Pseudomonadaceae
			k__Bacteria; p__Proteobacteria; c__Gammaproteobacteria; o__Enterobacteriales; f__Enterobacteriaceae; g__Yersinia; s__
			k__Bacteria; p__Proteobacteria; c__Gammaproteobacteria; o__Alteromonadales; f__OM60; g__Congregibacter; s__
			k__Bacteria; p__Verrucomicrobia; c__[Methylacidiphilae]; o__Methylacidiphilales; f__LD19; g__; s__
			k__Bacteria; p__Planctomycetes; c__Planctomycetia; o__Pirellulales; f__Pirellulaceae; g__Pirellula; s__

Table S2. Statistical analysis of mean relative abundance (%) ± SD of phyla and genera between groups. Statistical Analysis of Metagenomics Profiles (STAMP) software was used to test statistical significance between groups abundances, unclassified reads were retained only for calculating frequency. One-Way ANOVA (P<0,05), with an effect size (ETA-Squared) with Benjamini-Hochberg FDR correction was applied followed by Tukey-Kramer post-hoc test.

	**Autumn 2017**				**Summer 2018**				**p-values (corrected)**	**Effect size**
	**Pond**		**Lake**		**Pond**		**Lake**			
Phylum	mean abu.(%)	± SD	mean abu. (%)	± SD	mean abun. (%)	± SD	mean abu. (%)	± SD		
Verrucomicrobia	0.32	0.59	1.92	2.26	0.28	0.62	2.91	1.60	0.02677	0.42
Bacteroidetes	8.70	5.22	3.94	2.34	8.81	8.35	0.73	1.09	0.13059	0.31
Chloroflexi	0.00	0.00	0.25	0.34	0.00	0.00	0.00	0.00	0.10285	0.31
Planctomycetes	0.00	0.00	1.57	2.52	0.10	0.19	0.30	0.23	0.33806	0.21
Firmicutes	16.57	10.10	21.72	20.47	7.86	12.92	5.65	7.43	0.37285	0.19
Actinobacteria	6.50	6.37	9.71	10.04	3.61	7.36	0.86	0.68	0.34879	0.18
Proteobacteria	66.41	19.12	57.61	29.13	74.74	24.16	83.49	11.14	0.41028	0.16
Acidobacteria	0.91	1.93	1.79	1.97	0.59	1.32	0.25	0.25	0.61478	0.11
Synergistetes	0.03	0.06	0.00	0.00	0.00	0.00	0.00	0.00	0.57378	0.11
WS6	0.04	0.09	0.00	0.00	0.00	0.00	0.00	0.00	0.52162	0.11
Tenericutes	0.00	0.00	0.00	0.00	0.07	0.18	0.00	0.00	0.58042	0.09
Cyanobacteria	0.09	0.19	0.09	0.18	0.42	1.00	0.39	0.20	0.65605	0.07
Fusobacteria	0.36	0.62	0.30	0.42	2.80	7.02	1.49	2.48	0.66832	0.06
Genus										
Methylobacterium	38.88	19.08	0	0	0.09	0.24	0	0	0.00003	0.76
Candidatus Xiphinematobacter	0.01	0.03	0.03	0.04	0.05	0.13	2.01	1.02	0.00007	0.72
A17	0	0	0.15	0.14	0	0	0	0	0.05071	0.49
Desulfobulbus	0	0	0.11	0.11	0	0	0.18	0.14	0.05514	0.46
MSBL3	0	0	0.05	0.08	0	0	0.18	0.15	0.05505	0.45
Phycicoccus	0	0	0.13	0.13	0	0	0.01	0.02	0.06289	0.44
Aeromonas	10.05	14.86	17.38	17.71	47.93	38.14	62.61	18.94	0.07229	0.43
Desulforhopalus	0	0	1.87	1.9	0	0	0.38	0.24	0.08472	0.42
Yersinia	0	0	0.01	0.03	0	0	0.35	0.37	0.14502	0.38
Crenothrix	0	0	5.08	5.77	0	0	0.59	0.51	0.15432	0.37
Flavobacterium	1.84	2.18	0	0	5.28	5.16	0.03	0.08	0.15226	0.36
Congregibacter	0	0	0.1	0.13	0	0	0.31	0.32	0.15184	0.35
Dysgonomonas	0	0	0.73	0.9	0	0	0.01	0.02	0.1561	0.35
C39	0	0	0	0	0.55	0.65	0	0	0.17437	0.34
Tolumonas	0.06	0.14	0.14	0.27	0.54	0.53	0	0.01	0.17624	0.33
Modestobacter	0	0	0.27	0.35	0	0	0	0	0.20247	0.32
Allochromatium	0	0	0.51	0.68	0	0	0.03	0.05	0.21644	0.31
Chthoniobacter	0	0	0.31	0.41	0	0	0.02	0.04	0.21327	0.31
Paulinella	0	0	0	0	0	0	0.14	0.19	0.2234	0.3
Acidovorax	0.02	0.05	0.26	0.3	0.04	0.09	0	0.01	0.23668	0.29

Lactobacillus	0.52	0.59	0.21	0.42	0	0	0	0	0.26428	0.28
Propionibacterium	0.93	0.54	0.81	1.15	0.34	0.32	0.03	0.03	0.25333	0.28
Shewanella	0	0	0.84	0.82	0	0.01	0.8	1.1	0.30064	0.26
Luteolibacter	0	0	1.52	1.93	0.24	0.62	0.29	0.2	0.30448	0.26
Chitinibacter	0	0	0.04	0.08	0.1	0.19	0.27	0.27	0.30303	0.26
Deefgea	0	0	1.6	1.89	0.05	0.08	0.87	1.26	0.30532	0.26
Arenimonas	0	0	0	0	0	0	0.04	0.06	0.34298	0.24
Streptococcus	1.14	1.17	0	0	0.98	1.33	0.04	0.06	0.35122	0.24
planctomycete	0	0	0.62	0.98	0	0	0.05	0.07	0.36173	0.24
[Clostridium]	0.06	0.14	1.8	3.27	0.1	0.25	4.29	5.6	0.38117	0.23
Anaerococcus	0.09	0.15	0	0	0	0	0	0	0.42719	0.22
Prevotella	0.6	0.97	0.01	0.02	0	0	0	0	0.42028	0.22
Flavihumibacter	0.85	1.37	0.08	0.15	0	0	0	0	0.4381	0.22
Mannheimia	0	0	0	0	6.06	9.73	0.05	0.12	0.43764	0.22
Corynebacterium	3.68	5.68	1.17	1.75	0.07	0.1	0.01	0.02	0.44198	0.21
Bacillus	0.3	0.51	0	0	0	0	0	0	0.43602	0.21
Anaeromyxobacter	0	0	0.57	1.02	0	0	0	0	0.4405	0.21
Clostridium	0.46	0.97	4.47	6.86	0.67	1.22	0.29	0.19	0.4365	0.21
Rothia	0.21	0.36	0	0	0	0	0	0	0.46391	0.2
Clostridium 1	0	0	0.19	0.35	0	0	0	0	0.4782	0.2
Pseudomonas	7.78	13.38	0.76	0.8	0.4	0.46	0.14	0.13	0.52458	0.19
Haemophilus	0.57	1.05	0	0	0	0	0	0	0.53372	0.18
Lutimonas	0	0	1.09	1.92	0	0	0.41	0.56	0.55596	0.18
Xanthomonas	0	0	0	0	0	0	0.02	0.04	0.57052	0.18
Veillonella	0.21	0.33	0	0	0.08	0.14	0	0	0.56388	0.18
Achromobacter	1.35	2.37	0	0	0.22	0.49	0.01	0.01	0.5649	0.17
Pelosinus	0	0	0.14	0.29	0	0	0	0	0.57484	0.17
Geobacter	0	0	0.08	0.16	0	0	0	0	0.54645	0.17
Salinibacterium	0	0	0.19	0.38	0	0	0	0	0.53979	0.17
Neorickettsia	0	0	0.3	0.6	0	0	0	0	0.53329	0.17
Cellulomonas	0	0	0.05	0.11	0	0	0	0	0.52074	0.17
Solirubrobacter	0	0	0.04	0.08	0	0	0	0	0.50877	0.17
Carnobacterium	0	0	0.1	0.2	0	0	0	0	0.50299	0.17
Legionella	0	0	0.09	0.18	0	0	0	0	0.49181	0.17
Aeromicrobium	0	0	0.13	0.27	0	0	0	0	0.47594	0.17
Actinoplanes	0	0	0.16	0.32	0	0	0	0	0.45632	0.17
Pirellula	0	0	0.53	1.05	0	0	0	0.01	0.4554	0.17
Kocuria	0	0	1.16	2.32	0.07	0.19	0	0	0.47582	0.16
Bacteroides	1.34	1.74	0.46	0.9	0.45	1.17	0	0	0.48416	0.16
Cloacibacterium	0	0	1.27	2.54	0.12	0.31	0	0	0.4857	0.16
Acinetobacter	0.38	0.67	0	0	0.88	1.37	0.08	0.21	0.49159	0.16
Mycobacterium	0	0	0.19	0.38	0	0	0.06	0.04	0.49067	0.16
Azovibrio	0	0	0.12	0.24	0	0	0.02	0.03	0.49162	0.16
Rheinheimera	0	0	0	0	0.27	0.53	0	0	0.48602	0.15
Eikenella	0.44	1.09	11.77	23.5	10.04	12.94	0.42	0.5	0.48678	0.15
Pimelobacter	0	0	0.28	0.57	0	0	0.04	0.11	0.48867	0.15

Nostocoida	0	0	0.08	0.15	0	0	0.03	0.04	0.50824	0.15
Pantoea	0	0	1.27	2.55	0.35	0.57	0	0	0.50577	0.15
Micrococcus	0.22	0.35	0.74	1.48	0.04	0.1	0.01	0.01	0.5243	0.14
Staphylococcus	5.34	3.01	10.3	18.55	3.42	6.7	0.16	0.26	0.56351	0.13
u114	0.01	0.02	0.3	0.42	0.19	0.48	1.32	2.51	0.57408	0.13
Elizabethkingia	0.34	0.84	0.09	0.18	1.58	3.11	0	0	0.58422	0.13
Actinobacillus	0.13	0.32	0.63	1.27	0.1	0.25	0	0	0.58626	0.13
Arcobacter	0.11	0.18	0.12	0.24	0.03	0.07	0	0	0.62783	0.12
Paracoccus	0	0	0.02	0.04	0.03	0.08	0.08	0.12	0.63202	0.12
Stenotrophomonas	0.1	0.24	0.18	0.35	0	0	0	0	0.65154	0.12
Wandonia	0	0	0	0	0.24	0.56	0	0	0.64972	0.12
Vogesella	0.03	0.07	0	0	0.1	0.2	0	0	0.6557	0.11
Friedmanniella	0	0	0.08	0.17	0.04	0.1	0	0	0.6656	0.11
Neisseria	0.13	0.33	0	0	0	0	0	0	0.65271	0.11
Blastomonas	0.03	0.08	0	0	0	0	0	0	0.64791	0.11
Allobaculum	0.09	0.23	0	0	0	0	0	0	0.63852	0.11
Akkermansia	0.25	0.6	0	0	0	0	0	0	0.6294	0.11
Pseudoxanthomonas	0.52	1.27	0	0	0	0	0	0	0.62494	0.11
Sedimentibacter	0.13	0.31	0	0	0	0	0	0	0.62053	0.11
Ruminococcus	0.47	1.15	0	0	0	0	0	0	0.61619	0.11
Methylopila	0.05	0.12	0	0	0	0	0	0	0.61192	0.11
Pedobacter	0.12	0.29	0	0	0	0	0	0	0.6077	0.11
Oscillospira	0.06	0.16	0	0	0	0	0	0	0.59943	0.11
Porphyromonas	0.05	0.11	0	0	0	0	0	0	0.59538	0.11
rc4-4	0.06	0.14	0	0	0	0	0	0	0.59138	0.11
HA73	0.03	0.06	0	0	0	0	0	0	0.58744	0.11
Parabacteroides	0.53	1.29	0	0	0	0	0	0	0.58355	0.11
Blautia	0.05	0.13	0	0	0	0	0	0	0.57218	0.11
Selenomonas	0.11	0.26	0	0	0	0	0	0	0.56849	0.11
Bilophila	0.14	0.35	0	0	0	0	0	0	0.56485	0.11
02d06	0.06	0.15	0	0	0	0	0	0	0.55419	0.11
Syntrophus	0.1	0.25	0	0	0	0	0	0	0.55072	0.11
Peptoniphilus	0.04	0.1	0	0	0	0	0	0	0.5473	0.11
Actinomyces	0.06	0.14	0	0	0	0	0	0	0.53404	0.11
[Prevotella]	0.13	0.32	0	0	0	0	0	0	0.53082	0.11
Clostridium 2	0.35	0.86	0	0	0	0	0	0	0.52764	0.11
Polynucleobacter	0.63	0.98	0.01	0.03	0.85	1.79	0	0	0.52573	0.11
Dialister	0.38	0.94	0.02	0.02	0	0	0	0	0.53187	0.11
Leuconostoc	0.23	0.55	0.01	0.02	0	0	0	0	0.53114	0.11
Cylindrospermopsis	0.09	0.19	0	0	0.02	0.05	0	0	0.54514	0.11
Enhydrobacter	0.09	0.23	0.11	0.23	0	0	0	0.01	0.56204	0.1
Lactococcus	0.37	0.89	0.74	1.48	0.15	0.24	0	0	0.56079	0.1
Roseburia	0.04	0.09	0	0	0.01	0.02	0	0	0.57372	0.1
Dyadobacter	0	0	0	0	0.04	0.12	0	0	0.59095	0.09
Alistipes	0	0	0	0	0.07	0.19	0	0	0.58771	0.09
Roseococcus	0	0	0	0	0.32	0.84	0	0	0.5845	0.09

Flectobacillus	0	0	0	0	0.14	0.37	0	0	0.58132	0.09
Candidatus Hamiltonella	0	0	0	0	0.22	0.59	0	0	0.57199	0.09
Sulfurospirillum	0	0	0	0	0.07	0.2	0	0	0.56895	0.09
Aquabacterium	0	0	0	0	0	0	0.01	0.02	0.56296	0.09
Serratia	0	0	0	0	0	0	0.07	0.19	0.5571	0.09
Peredibacter	0	0	0	0	0.03	0.08	0	0	0.55135	0.09
Cetobacterium	0.35	0.62	0	0	2.61	6.53	0.17	0.13	0.55328	0.09
Morganella	0.01	0.02	0	0	0	0	0.16	0.42	0.55447	0.09
Sediminibacterium	0.51	1.24	0.01	0.01	0.19	0.39	0	0	0.55805	0.09
Chitinilyticum	0	0	0	0	0.02	0.04	0.02	0.04	0.56265	0.09
Enterococcus	0	0	0.02	0.05	0.06	0.17	0	0	0.57218	0.08
Desulfovibrio	0	0	0	0	0.33	0.87	0.16	0.18	0.59531	0.08
Methylocaldum	0	0	0.33	0.58	0.64	1.6	0.36	0.32	0.67444	0.06
